# Behavior of Lung Health Parameters among Smokers and Secondhand Smokers

**DOI:** 10.1155/2018/5217675

**Published:** 2018-03-04

**Authors:** Esther Ghanem, Re-Mi Hage

**Affiliations:** ^1^Department of Sciences, Faculty of Natural and Applied Sciences, Notre Dame University, P.O. Box 72, Zouk Mosbeh, Lebanon; ^2^Department of Mathematics and Statistics, Faculty of Natural and Applied Sciences, Notre Dame University, P.O. Box 72, Zouk Mosbeh, Lebanon

## Abstract

A cross-sectional study on a pool of undergraduate smokers and nonsmokers (*n* = 200) was randomly selected from Notre Dame University, Lebanon. The study design is based on a questionnaire about the students' smoking record exposure, cotinine saliva levels, and ventilatory lung function parameters. Despite the nonsmoking policies that have been recently established by universities, diffused smoking stations in proximity to classes and offices still exist, at least in the MENA region. Such an environment still imposes a remarkable effect on certain lung health parameters of nonsmokers exhibiting similar exhaled air per second (FEV1) to smokers with a *P* value = 0.558 and normal flow of air (TV) with a *P* value = 0.153. However, the maximum amount of air held in the lungs remained different with respect to sex and smoking status. These results imply a poor performance of nonsmokers mimicking partially the lung health parameters of smokers. It remains a pressing issue to increase awareness concerning the debilitating effects of secondhand smoking.

## 1. Introduction

Cigarette smoking is a serious international health concern with a spectrum of health risks that is mainly stemming from a chronic nicotine addiction at an early onset in young adults [1, 2]. Despite the extensive media on the harmful effects of tobacco, the rules and regulations that aim to reduce smoking, and the extensive research documenting the side effects of secondhand smoke (SHS) exposure among nonsmoking adults, the number of smokers is steadily rising in different parts of the world, from Southeast Asia to the USA [3–5]. Lebanon is not doing well too as compared to other countries: it is estimated that more than a third of the Lebanese adult population smokes, with one of the highest tobacco consumption rates in the MENA region [6].

The real rate of environmental tobacco smoke (ETS) exposure, whether active or passive, can be determined by measuring saliva cotinine levels [7]. The exact impact of smoking on health can be evaluated by correlation with accurate ETS exposure rates. Smoking has been shown to critically alter pulmonary and cardiovascular function and is closely linked to chronic diseases in those systems [8]. Pulmonary ailments can be detected by measuring a series of respiratory setpoints, namely, the vital capacity (VC), the tidal volume (TV), and the ratio of forced expiratory volume in one second (FEV1) to vital capacity (FEV1/VC). The VC is the maximal amount of air which can be held within the lungs. TV is the amount of air that is normally inhaled and exhaled during a regular breathing cycle. The FEV1/VC ratio is used to diagnose obstructive and restrictive lung diseases. Previous studies have shown that smoking causes a decrease in the values of the abovementioned parameters, which indicates deficits in respiratory function [4, 5, 9–11].

A spectrum of research has been reported to evidence smoking-related health deterioration and to encourage smoking cessation in different regions across the globe [12], from India [4, 11] to Thailand [5], extending to Lebanon. However, reports about Lebanon mainly have economic or sociodemographic approaches rather than scientifically based approaches [6, 13–15]. Moreover, studies testing the effect of SHS exposure on the lung health function parameters have been already conducted in children [16–19], adolescences [20], adults [21], but not yet in young adults. There is only one study mainly associated with the level of cigarette consumption per day in young adults and the development of respiratory symptoms [22]. Information from the literature demonstrates a reduction in the lung function parameters among adolescences who smoke or are exposed to SHS and more susceptibility for both groups to develop respiratory problems including cough, phlegm, asthma, and wheezing [23–26].

Hence, comparing the lung function of SHS exposed students to those of smokers in young adults is necessary to expand the body of evidence of SHS deteriorating effects

## 2. Methods

### 2.1. Setting and Participants

The study was conducted over a 3-month period extending from April to June 2015 at NDU main campus. Measurements did not overlap with the final exam period to exclude any stress factor. Participants were asked to fill a questionnaire covering the sex, age, Body Mass Index (BMI), years of smoking, daily nicotine consumption, the overall level of fitness, and the location of exposure to secondhand smoking at NDU.

The total number of valid respondents was 200 (excluding students with BMI < 18 or >25). The effect of BMI on the pulmonary function test (PFTs) has been widely investigated in the literature [27–30]. For instance, low BMI (underweight) has been documented to lower the PFT values (FEV1, FVC) for both smoker and nonsmokers compared to normal individuals, whereas high BMI (overweight) showed significantly lower FVC compared to normal individuals [31]. Thus, to exclude the effect of low and high BMIs on our readings, only fit individuals participated in the project.

Age ranged from 18 to 24 years with a mean of 20 years old and a standard deviation of 1.74 years old. The BMI ranged between 17.79 and 25.83 with an average of 21.39 and standard deviation of 2.38. None of the participants showed physical or health disorders at the recording time period. Sex participants included 40% female and 60% male, out of which 47% are nonsmokers and 53% are smokers.

### 2.2. Ethics Statement

This study is in compliance with the recognized international standards and principles of the Declaration of Helsinki and has received ethical approval from the institutional review board (IRB) at Notre Dame University with the following protocol number: IRBF16_2_FNAS.

### 2.3. Lung Inhalation and Exhalation Capacity

The spirometer tests were collected at the Biology Laboratory at NDU using the PASCO Spirometer GLX model, PS-2152, by P. K. Morgan Medical Lab. Spirometer disposable mouthpieces were purchased from PASCO, PS-2522.

Subjects were asked to stand or sit comfortably in front of the machine. With closed nose, four normal breaths using the mouthpiece were taken. After which, one deep inhalation was taken followed by a forced expiratory breath and two normal breaths were recorded. Pulmonary function tests (PFTs) complied with the conventional guidelines adopted by the British Thoracic Society [32] given the complexity and lengthy manual provided by the ATS [33].

Pulmonary function parameters were analyzed using Data Studio version 1.9.8r9 from flow-volume loops spirograms that depict the rate of airflow on the *y*-axis and the total volume of air, inhaled or expired, on the *x*-axis.

The following values in liter (L) were used as healthy lung function parameters based on sex category:

- female with VC > 3.2, FEV1 > 2.7, FEV1/VC > 0.8, and TV > 0.4;
- male with VC > 3.4, FEV1 > 2.4, FEV1/VC > 1, and TV > 0.5.

### 2.4. Saliva Nicotine Tests

Saliva cotinine levels serve as an accurate assessment means of students' exposure levels to cigarette smoke [34, 35]. Cotinine is the main metabolite of nicotine, and its presence in saliva and serum is almost exclusive to previous tobacco exposure [36, 37]. There is a good correlation between saliva and plasma cotinine concentrations [7]. Cotinine has a half-life of 15–40 hours, so it is effective in detecting secondhand smoking within 2-3 days [31].

The amount of cotinine in the saliva of participants was measured using the NicAlert saliva nicotine test, purchased from Craig Medical Distribution Inc., CA, USA. Sufficient amount of saliva was deposited in a special container through a funnel. Eight drops of saliva from each sample were poured on the tip of a NicAlert test strip.

### 2.5. Statistical Method

Data were entered, edited, and analyzed using Matlab R 2013a. Independent sample *t*-test was used for the parameters TV, VC, FEV1, and FEV1/VC in comparison with sex and smoking status. Analysis of Variance, one-way ANOVA, was used to compare means of sex and smoking status combined together with respect to the above parameters. Finally, chi-square test was used to test the independency between the parameters and the variables.

### 2.6. Limitations of the Study

Time and funds allocated for the completion and execution of the study were not adequate to sample enough data. Moreover, there was no access to the medical files of the participants, and information collected was based on the participants' feedback. This might have produced some inaccuracy in our recorded health parameters. In addition, given the spirometer sensitivity limit, data collection may be subject to fluctuations based on the proper handling of the machine by the operating personnel or certain unnoticeable air pressure environmental changes.

## 3. Results

Despite the young age of NDU undergraduate students (mean age 20 years), 47% of the tested pool exhibited a nonsmoking profile, whereas almost 38% of the smokers have been smoking for at least 3 years. To assess the effect of secondhand smoking, saliva cotinine levels (mg/ml) were measured for nonsmokers. Only 15.8% of nonsmokers have 0 ng/ml cotinine and at least 50% have between 10 and 30 ng/ml cotinine in their saliva, thus making evidence of exposure to secondhand smoking. The virulent fragment in the smoking profile lies in the behavior of the majority, almost 23% of smokers as light smokers with a range of saliva cotinine levels of [10–30 ng/ml].

Moreover, the saliva cotinine profile of smokers displayed the positioning of the majority of smokers in a medium category with cotinine values of [100–500 ng/ml] and approximately 25% as heavy smokers (data not shown).

**Figure 1:**
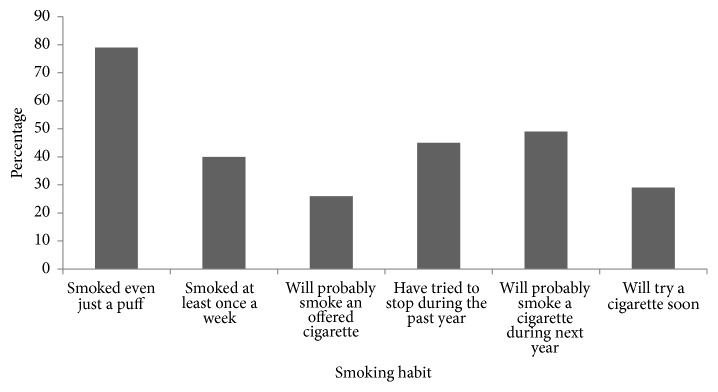
Bar graph represents the percentage of students who showed a variation in their smoking habit and trial as collected from a survey conducted during the spring semester 2015.

As a matter of fact, the society and the entourage seem the primary factors that trigger a nonsmoker to become a light smoker. Based on data collected from a survey conducted throughout the spring semester 2015, as represented in Figure 1, we can notice that almost a third of the students (26%) smoked an offered cigarette, 79% tried few puffs, 49% will probably smoke a cigarette during the next year, and 29% are willing to try a cigarette soon.

To further assess the effects of secondhand smoking on the lung function of nonsmokers, selected lung parameters, namely, the VC, TV, and FEV1/VC, were tested and compared with respect to sex, smoking status, and both variables combined.  Table 1 represents the frequency, mean, and standard deviation of each of the criteria with respect to sex. Interestingly, all the parameters, with the exception of FEV1, showed difference of means with respect to sex with *P* values < 0.05.

**Figure 2:**
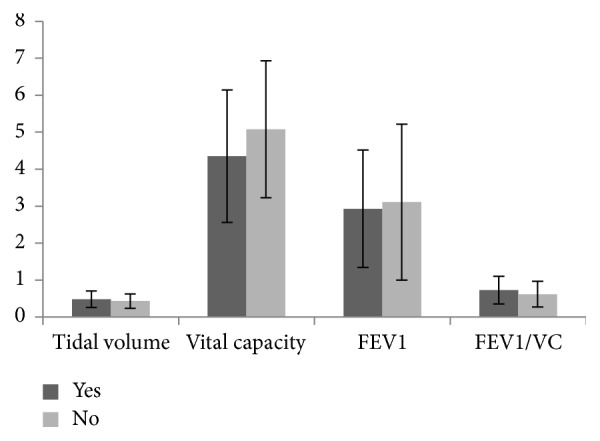
Bar graph representing the mean and standard deviation of TV, VC, FEV1, FEV1/VC with respect to the subjects' smoking status.

Using the same statistical method, we examined the pulmonary parameters in rapport with the smoking status.  Figure 2 represents the frequency, mean, and standard deviation of each of the tested pulmonary parameters with respect to smoking.

To identify the significant difference between the means of the tested parameters, again we reverted to the *P* value of VC and FEV1/VC which scored 0.025 and 0.048, respectively, pointing to a significant variation of these values with respect to the smoking status. On the other hand, TV and FEV1 did not vary regardless of the smoking status as indicated by their *P* values of 0.153 and 0.558, respectively.

Thus, only TV and FEV1 show similarity regarding the smoking status. So far, FEV1 values did not significantly vary neither by sex nor by the smoking status.

### 3.1. Assessing the Combined Effect of Sex and Smoking Status

We further characterized using ANOVA to compare the tested parameters in rapport with the two combined variables, sex and smoking. *P* values of TV and VC are less than 0.05, supporting our claim that these two parameters differ with respect to sex and the smoking status (Table 2).

It is worth noting that even combining sex with smoking status did not affect FEV1 and FEV1/VC values among smokers and nonsmokers.

We then computed the Pearson chi-square to test whether the selected lung parameters (healthy/unhealthy described in research method) are independent from sex, smoking, and both pooled together (Table 3). It was again clearly noticeable that FEV1 is independent from sex, smoking, and both variables (Sex/Smoking) combined together.

## 4. Discussion

Driven by the bad influence of smoking on our health and society, we simulated a case study to assess the effect of secondhand smoking exposure on selected pulmonary function parameters. Similar studies have been reported in the literature, but not yet on a Lebanese pool of students neither on secondhand smoking exposure. Thus, it was intriguing to conduct and translate such a study in Lebanon. NDU arena served as a platform given the radical increase in the prevalence of smoking, especially those who join the smokers crew for the sake of fun or following a trend as retrieved by our questionnaires.

Smoking among young NDU adults demonstrated subtle side effects on certain pulmonary parameters in both sexes, mainly FEV1 that lied within the healthy conditions while exhibiting intricate consequences on FEV1/VC that lied within unhealthy category. VC and TV showed healthy averages of values corresponding to 3.9 and 5.3, and 0.4 and 0.5 for both females and males, respectively. However, their means varied in light of sex and only showed a difference for VC in light of the smoking status. Such results reflect the role that secondhand smoking plays in shaping the volume of air that can be expelled by the lungs after a deep breath.

Interestingly, FEV1 showed healthy values with an average of 2.9 and 3.1 for smokers and nonsmokers, respectively. Our data diverge from what was previously reported for lower FEV1 values of 2.68 and 2.96 for smokers and nonsmokers, respectively [5]. In addition, females with FEV1 of 2.8 are more susceptible to develop lung disorders given the slight increase above the limit of healthy condition (>2.7) compared to men with FEV1 of 3.1 which is quite higher than the limit (>2.4). Moreover, the ratio FEV1/VC was dependent on sex with an average of 0.75 and 0.6 for females and males, respectively. Both values lie within the unhealthy category with male smokers showing more deviation from the predicted value since the difference between 0.6 and 1 is higher than the difference of female smokers between 0.7 and 0.8, whereby 0.8 and 1 are predicted values of healthy subjects. We could attribute this to the dominant male sex among the tested pool.

Comparing our results with previous lung function parameters from group of adults aged 20–25 years [11], we assume that the normal values of TV and VC on one hand and the reduced values of FEV1/VC below their relevant predicted values might be consistent with an early risk of obstructive lung diseases, such as airway narrowing during exhalation, asthma, emphysema, and chronic bronchitis [22].

Overall, nonsmokers behaved like smokers in their performance to exhale air per second (FEV1) and in the normal flow of air per breath (TV). This implies a poor performance that is most likely caused by secondhand smoking without excluding the effect of other factors, such as a possibility of weak physical education.

Saliva cotinine in nonsmokers was aligned with what was previously reported, pointing to the overlooked effect of secondhand smokers in the society [35]. Nonsmoking students, who showed devastating reactions to smokers and/or emitted nicotine, were surprised to show a level 1 or 2 smoking with values range of [10–100] ng/ml. NicAlert cotinine has been compared to GC saliva measurements and has been shown to be a valid and reliable test [38]. Thus, our finding that nonsmokers do exhibit nicotine in their saliva and do act as mild smokers is quite reliable.

Doing simple calculation, we assessed the effects of cigarettes litter on the environment. If an estimate butt produces 9 mg to 30 mg nicotine depending on the type of cigarette, then on-campus, the 3000 collected butts in one week would result in an average of ~27 to 90 g of weekly nicotine. This projects not only the large amount of nicotine produced in the atmosphere, but also the tremendous emission of other carcinogenic chemicals. Nonetheless, one cannot neglect the fate of cigarette butts disposal that is detrimental to the overall health of our ecosystem threatening plants, soils, and animals. Hence, policy interventions and legislation and overall awareness should be highly raised to advocate against smoking starting from university campuses followed by houses and public environment. Secondhand smoking is contagious and mobilizes a set of anomalies in our physiological systems, namely, the respiratory, immune, and nervous systems. Our results pave the way to further explore the debilitating effects of secondhand smoking on a larger pool of students from different local and regional territories as a means to increase awareness and support action research in health promotion.

## 5. Conclusion

Many studies have been conducted to evidence smoking-related health deterioration and to encourage smoking cessation in different regions across the globe. Observing the wave of nicotine addiction especially among undergraduate students as a means to socialize and become productive is exponentially becoming trendy and out of control. The best way to raise awareness is to run a study among students and monitor their lung function via simplistic methods, such as nicotine saliva tests and mouthpiece spirometers. Despite the nonsmoking policies that have been recently implemented at the campuses of different universities, diffused smoking stations in proximity to classes and offices still exist. Such an environment still imposes a remarkable effect on certain lung health parameters of nonsmokers, especially on the total amount of expelled air per second (FEV1) and TV. These results imply a poor performance of nonsmokers mimicking partially the lung health parameters of smokers. It remains a pressing issue to increase awareness concerning the debilitating effects of SHS and the need to apply stringent regulations to the implementation of the no-smoking policy within the premises of universities.

## Figures and Tables

**Table 1:** The frequency, mean, and standard deviation of TV, VC, FEV1, FEV1/VC with respect to sex. *P* values are also depicted with 95% confidence interval.

	Sex	Frequency	Mean	Standard deviation	*P* value	95% confidence interval of the difference	
						Lower	Upper
TV	Female	47	0.401	0.191	0.008	−.167	−.0254
	Male	103	0.497	0.222			
VC	Female	47	3.923	1.572	0.001	−1.928	−.769
	Male	103	5.272	1.834			
FEV1	Female	47	2.850	1.950	0.391	−.980	.387
	Male	103	3.146	1.964			
FEV1/VC	Female	47	0.750	0.393	0.038	.008	.271
	Male	103	0.611	0.334			

**Table 2:** *P* values of the ANOVA test for the combined variables: sex and smoking.

Parameters	Speed (rpm)
TV	0.042
VC	0.001
FEV1	0.802
FEV1/VC	0.117

**Table 3:** *P* values to test the independence of the lung parameters from sex, smoking, and both variables combined.

Parameters	Sex	Smoking	Smoking/sex
TV	0.015	0.758	0.048
VC	0.001	0.001	0.002
FEV1	0.627	0.896	0.77
FEV1/VC	0.018	0.042	0.057

## Data Availability

The datasets used and/or analyzed during the current study are available from the corresponding author on reasonable request.
